# New Insights into Pathogenesis, Diagnosis, and Treatment of Pancreatic Disorders

**DOI:** 10.1155/2013/305179

**Published:** 2013-02-17

**Authors:** Grazyna Rydzewska, Jean Morisset, Davor Stimac

**Affiliations:** ^1^Gastroenterology Department, Central Clinical Hospital of Ministry of Internal Affairs, Warsaw, Poland; ^2^The Jan Kochanowski University in Kielce, 25-369 Kielce, Poland; ^3^Sherbrooke University, Sherbrooke, QC, Canada J1K 2R1; ^4^Division of Gastroenterology, University Hospital Rijeka, Krešimirova 42, 51000 Rijeka, Croatia

Pancreatic disorders, such as acute and chronic pancreatitis and pancreatic cancer, are until now challenging diseases to manage, with significant burdens of morbidity, mortality rate, and high financial costs. Acute pancreatitis (AP) is still a common and potentially fatal disease with nonspecific treatment and unpredictable prognosis. In spite of significant improvement in understanding the basic pathophysiology of the disease and advances in the diagnosis and management of acute pancreatitis, the mortality rate has remained stable since the 1970s. Chronic pancreatitis (CP), resulting in a slow irreversible damage of the organ associated with gradual declines in digestive enzyme production, is the major cause of pancreatic exocrine insufficiency in adults. Finally, pancreatic cancer is known as a devastating process with only 6-month median survival after diagnosis.

 In the present special issue,some recent advances in research concerning pancreatic diseases are presented. Croatian authors discussed epidemiology of AP in the North Adriatic Region of Croatia. They showed epidemiological characteristics typical of Mediterranean countries with predominant biliary etiology. 

Early prediction of severity of acute pancreatitis (AP) by a simple unique laboratory test would be very helpful as it could direct management from the very beginning and at the end improve the outcome. Commonly accepted scales estimating the AP severity are extremely hard to use in clinical practice. For this reason, it is understandable why further attempts to find a parameter or a set of parameters useful in diagnosis and prognosis in AP are so necessary. Despite the importance of early prognosis, many patients initially identified as having a mild disease progress to severe acute pancreatitis (SAP) over time. Many biochemical parameters (including C-reactive protein (CRP), procalcitonin, and trypsinogen activation peptide (TAP)) have been evaluated in the severity assessment of AP. 

In the present issue, authors discussed prognostic value of interleukins (IL) 6, 8, and 10, soluble receptor for tumor necrosis factor (sTNFr), pancreatic elastase (E1), and C-reactive protein (CRP) as predictors of systemic complications in patients with AP. They found that IL-6, IL-8, IL-10, and sTNFr measured on admission and CRP and pancreatic elastase measured on the third day of admission represent valuable prognostic factors in the determination of severity and systemic complications in patients with AP. Recently, parameters showing distortion of water balance in the course of AP have been a subject of research and clinical observations and were found very promising in severity and death prediction.

Although the pathogenesis of post-ERCP pancreatitis is not clearly understood, it seems that the patient's inflammatory response to pancreatic duct imaging and instrumentation plays a critical role. Several studies pointed out to special factors, which put an individual in high risk for the development of post-ERCP pancreatitis. The history of post-ERCP pancreatitis as an independent risk factor for a new episode of post-ERCP pancreatitis seems to be very important. On the other hand, some individuals can have a genetically predisposed susceptibility for this particular complication. However, in the article published in this issue, the authors were not able to associate the role of SPINK1N34S mutation with post-ERCP pancreatitis. 

Pancreatic cancer (PC) is one of the cancers of limited occurrence; however, worldwide over 200000 people die annually of this disease. The highest incidence and mortality rates of pancreatic cancer are found in developed countries. In the United States, pancreatic cancer is the fourth leading cause of cancer death, and in Europe it is the sixth. PC occurs three times as often in high-income countries as in those of middle and low income. Therefore, understanding the etiology and identifying the risk factors remain essential for the primary prevention of this disease. The risk factors, which can be easily eliminated, include tobacco smoking, obesity, and diabetes mellitus. The extent to which diet affects pancreatic cancer risk is still unclear.

The authors of the article included in the present issue have shown a correlation between PC and tobacco smoking (0.55 and 0.44). They also discussed that high consumptions of fats, sugar, and alcohol are potential risk factors for PC. Therefore, they suggest that positive changes in the diet such as lowering red meat consumption and increasing fruit consumption could influence incidence and reduction in future years.

Furthermore, other investigators discussed some interesting data concerning some microenvironmental elements, which could be involved in the development of pancreatic cancer. It is well known that growth of dense, collagen-rich, extracellular matrix and stroma, known as the desmoplastic reaction, creates a unique microenvironment promoting both tumour growth, and metastatic spread and forms a barrier to specific chemotherapy drug penetration. The authors discussed new developments in the fight against desmoplastic reaction, including inhibitors of the epidermal growth factor, fibroblast growth factor as well as new molecular targets like CD40 agonist and their effects on T cells, extracellular matrix modifying enzymes such as LOXL2 inhibitor, and novel tumor-penetrating peptides for delivery of drugs. 

A group of scientists also described the involvement of macrophages in angiogenesis, process tumor growth and invasion of cancer cells. Macrophages are the source of angiogenic factors like VEGF and also MMP9, which degrade extracellular matrix. Therefore, macrophages infiltrating pancreatic tumor are another important factor promoting metastases. It was established that among many factors influencing tumor microenvironment c-Met receptor, infiltrating macrophages and MMP2 have significant influence on the development and invasion of pancreatic cancer.

Endoscopic ultrasound-guided fine needle aspiration (EUS-FNA) is now considered as one of the most useful methods for histological diagnosis and staging of pancreatic cancers. The Japanese discussed the usefulness, cost-effectiveness, and accuracy of EUS-FNA as a diagnostic modality for evaluating pancreatic tumors. They also underlined the role of molecular biological analyses for the diagnosis of PC. Other authors showed the utility of percutaneous ultrasonography-guided biopsy as a diagnostic tool for PC; however, it seems that this method should be rather reserved for nonresectable tumors.

We trust that the data presented in this special issue will help in understanding pancreatic disorder pathogenesis, especially microenvironment elements involved in the development of pancreatic cancer tumor. This hopefully will give new ideas for the development of new therapeutic strategies in pancreatic disorders.

*Grazyna Rydzewska* *Grazyna Rydzewska* *Jean Morisset* *Jean Morisset* *Davor Stimac* *Davor Stimac*

